# Y chromosome in health and diseases

**DOI:** 10.1186/s13578-020-00452-w

**Published:** 2020-08-13

**Authors:** Yun-Fai Chris Lau

**Affiliations:** 1 Division of Cell and Developmental Genetics, Department of Medicine, San Francisco VA Health Care System, University of California, San Francisco, 4150 Clement Street, San Francisco, CA 94121 USA; 2 Institute for Human Genetics, University of California, San Francisco, San Francisco, USA

**Keywords:** Y chromosome, Mosaic loss, Pseudoautosomal region, Male-specific region, Gene functions

## Abstract

Sex differences are prevalent in normal development, physiology and disease pathogeneses. Recent studies have demonstrated that mosaic loss of Y chromosome and aberrant activation of its genes could modify the disease processes in male biased manners. This mini review discusses the nature of the genes on the human Y chromosome and identifies two general categories of genes: those sharing dosage-sensitivity functions with their X homologues and those with testis-specific expression and functions. Mosaic loss of the former disrupts the homeostasis important for the maintenance of health while aberrant activation of the latter promotes pathogenesis in non-gonadal tissues, thereby contributing to genetic predispositions to diseases in men.

## Sex differences and mosaic loss of Y chromosome

Sex differences are prevalent in development and physiology in humans, and pathogeneses of various diseases, such as cancers, neurodevelopmental, neurodegenerative and cardiovascular diseases [1–8]. Such sex differences in diseases include incidence, onset age, progression, phenotypes and treatment responses. Although sex hormones, i.e. androgens and estrogens, and their receptors, i.e. androgen and estrogen receptors, could play important roles in such biological processes [1, 3], genes on the male-specific Y chromosome could also contribute at the genetic levels to such differences between the sexes [9–11]. Recent studies on large populations with well-defined health information suggest that mosaic loss of the Y chromosome (mLOY) in the peripheral white blood cells predisposes men to various diseases, including cancers, cardiovascular and Alzheimer’s diseases [12–17], suggesting that the Y chromosome could be essential in maintaining the homeostasis important for the health of men [11]. Despite such associations, it is uncertain how mLOY in small portion of the leukocytes could contribute to the genetic predisposition to diseases in men. One hypothesis suggests that such mLOY in peripheral white blood cells is an indicator of genome instability in general, affecting genes in lymphocytes and other tissues and/or various physiological processes, such as inflammation, immunosurveillance and oxidative stresses [12, 13, 18], with functional consequences across diverse biological systems. Indeed, various novel and known loci in cell cycle regulation and cancer susceptibility have been associated with mLOY [12, 13, 19], thereby supporting the essential nature of the Y chromosome genes in the well-being of men. Mosaic LOY disrupts such balances and predisposes men to pathogenesis, resulting in sex differences in the various diseases.

## Aberrant activation of Y chromosome genes potentiates pathogeneses of human diseases

Studies on individual Y chromosome genes have provided information suggesting that they could exert positive actions on the pathogeneses of various human diseases, including cancers and neurodevelopmental diseases, thereby preferentially affecting males [6, 20]. For example, expression of the sex-determining region Y (*SRY*) gene is elevated in dopamine neurons in human and experimental Parkinson’s disease models and suppression of its expression could exert protective functions against the disease in these models [21]. Studies on the spontaneously hypertensive rat (SHR) indicated that the rat *Sry* up regulates genes in the renin-angiotensin system, resulting in higher blood pressure in the male SHRs [22]. The human *SRY* could upregulate the monoamine oxidase A (*MAOA*) gene, whose expression levels are associated with various neurological and psychological disorders [23]. Further, aberrant expression of *SRY* could compete against the proper functions of a family of related transcription factors, encoded by the SRY-box (SOX) genes, which play critical roles in numerous developmental, physiological and pathogenic processes [24–26]. Indeed, *SRY* could impair the *SOX10* regulation of the *RET* gene, important for enteric nervous system (ENS) differentiation [6]. Such impairment results in haploinsufficiency of the RET protein and exacerbation of the pathogenesis of the Hirschsprung’s disease, a congenital disorder affecting the ENS differentiation with significantly high male preference [6]. Importantly, aberrant activation of a human *SRY* transgene during embryogenesis in transgenic mice impairs the normal development of various vital organs, resulting in postnatal growth retardation and lethality [27]. Hence, these studies suggest that aberrant activation of Y chromosome genes, in this case *SRY*, could disrupt normal development and exacerbate the disease processes, thereby contributing to sex differences in a positive manner(s). These findings are in contrast to those of mLOY studies, in which loss of genes on the Y chromosome potentiates the disease processes [13, 15, 18, 19]. To understand the roles of the Y chromosome in human health and diseases, it is crucial to discuss a few basic aspects of the genetics and biological functions of the genes on this male-specific chromosome.

## Genes on the human Y chromosome

In humans, men and women are genetically identical with 22 pairs of autosomes, except their sex chromosomes, i.e. X and Y chromosome. Men possess XY while women possess XX sex chromosome constitution. The gene dosage differences for the X chromosome is compensated by inactivation of the genes, with specific exceptions, on one of the X chromosomes during early development in females [28–30]. The mammalian X and Y chromosome originated from a pair of autosomes. One of them had acquired a male sex-determining gene, i.e. *SRY*, postulated to occur over 180 million years ago, and became the Y chromosome while the other homologue maintained the genetic content and structure of the ancestral chromosome and evolved to be the X chromosome [31]. Various evolutionary rearrangements and deterioration events resulted in a reduction in genetic content on the Y chromosome, which evolved to be the smallest and/or most gene-poor chromosome in most mammals, including humans [32]. The human Y chromosome is 57.23 MB in size and harbors two specific regions, generally referred to as the pseudoautosomal regions (i.e. PAR1 and PAR2) and male-specific region Y (MSY). PAR1 and PAR2 harbor ~ 2.6 MB and 320 kb of DNA and are located at the telomeric ends of the short and long arm of the Y chromosome respectively. The MSY is about 54 MB in size, consisting of approximately 24 MB of euchromatin and 30 MB of heterochromatin composed of mostly repetitive sequences (Genome Reference Consortium Human Build 38.p13). Cytogenetically, the heterochromatic long arm is highly variable, mostly involving either amplification or deletion of the heterochromatin and possibly the PAR2 at the telomere of the long arm [33, 34]. At present, there are 40 protein-coding genes/gene families on the human Y chromosome (Table 1) [35]. Fifteen genes are on the PAR1 and 3 genes are on the PAR2, which are also present in the PARs of the X chromosome. There is an obligatory crossover(s) between the X and Y chromosomes at their PARs during male meiosis [36], and hence genes on PARs behave similarly as those of the autosomes. The PAR genes escape X-inactivation and are mostly expressed in similar levels in both female and male tissues/cells [28] (Additional file 1: Figure S1A). Interestingly, there are 5 receptor genes, i.e. *CRLF2*, *CSF2RA*, *IL3RA*, *P2RY8* and *IL9R*, among the PAR genes, involved in various cytokine and immune functions. Initial sequencing results suggested that there are 78 protein-coding genes on the MSY, corresponding to 27 distinct proteins [10]. This number varies due to microdeletions and copy number variations (CNV) of ampliconic genes among the general population [37]. There are 22 MSY genes, of which 17 are evolutionarily conserved with corresponding X homologues, widely expressed (Additional file 1: Figure S1B-C) and postulated to serve dosage-sensitive regulatory functions in chromatin modification, transcription, translation, RNA splicing and protein stability [9, 10], which likely exert global effects on gene expression and modification of protein functions. Gene ontology analysis of all 40 Y chromosome genes suggested that they could be associated with male fertility/infertility, autism, coronary and psychological/neurological diseases (Additional file 1: Table S1).

**Table 1:** Protein-coding genes and functions on the human Y chromosome

Gene ID	Gene symbol	Gene name	Chr location (order:Yp - Yq)	Mouse ortholog (Chromosome)	Gene description^a^
55344	PLCXD1	Phosphatidylinositol specific phospholipase C X domain containing 1	Yp11.32-p11.31	Plcxd1 (chr 5)	A protein-coding gene at the most terminus of PAR1, encoding an enzyme catalyzes the formation of 1,4,5-trisphosphate and diacylglycerol from phosphatidyinositol 4,5-bisphosphate, involved in intracellular transduction of extracellular signals
8225	GTPBP6	GTP binding protein 6 (putative)	Yp11.31	Gtpbp6 (chr 5)	A PAR1 gene coding for a GTP binding protein, potentially associated with verbal ability and cognition
28227	PPP2R3B	Protein phosphatase 2 regulatory subunit B” beta	Yp11.31	Ppp2rla (chr 17)	A PAR1 gene encoding the protein phosphatase 2, one of the four major Ser/Thr phosphatases, likely inovlved in the negative control of cell growth and division
6473	SHOX	Short stature homeobox	Yp11.2	Shox2 (chr 3)	A PAR1 gene encoding a protein of the paired homeobox family, defects in this gene are associated with idiopathic growth retardation and in the short stature phenotype of Turner syndrome patients
64109	CRLF2	Cytokine receptor like factor 2	Yp11.2	Crlf2 (chr 5)	A PAR1 gene encoding a member of the type I cytokine receptor family and a receptor for thymic stromal lymphopoietin (TSLP), associated with acute lymphoblastic leukemia and Down syndrome
1438	CSF2RA	Colony stimulating factor 2 receptor subunit alpha	Yp11.2	Csf2ra (chr 19)	A PAR1 gene encoding for the alpha subunit of the heterodimeric receptor for colony stimulating factor 2 (CSF2), a cytokine controlling the production, differentiation, and function of granulocytes and macrophages; and associated with Surfactant metabolism dysfunction type 4
3563	IL3RA	Interleukin 3 receptor subunit alpha	Yp11.2	Il3ra (chr 14)	A PAR1 gene encoding an interleukin 3 specific subunit of a heterodimeric cytokine receptor; and a leukemia-associated antigen (CD123) highly expressed in leukemic stem cells and blasts
293	SLC25A6	Solute carrier family 25 member 6	Yp11.2	Slc25a5 (chr X)	A PAR1 gene encoding a member of the mitochondrial carrier subfamily of solute carrier proteins, functions as a gated pore for translocation of ADP from the cytoplasm into the mitochondrial matrix and ATP from the mitochondrial matrix into the cytoplasm, likely involved in the permability transition pore complex and apoptosis
8623	ASMTL	Acetylserotonin *O*-methyltransferase like	Yp11.2		A PAR1 gene encoding a protein with an N-terminus similar to the multicopy associated filamentation (maf) protein of Bacillus subtilis and to orfE of E. coli and a C-terminus similar to *N*-acetylserotonin *O*-methyltransferase
286530	P2RY8	P2Y receptor family member 8	Yp11.2	P2yr4 (chr X)	A PAR1 gene encoding a member of the family of G-protein coupled receptors. P2RY8 is frequently mutated in germinal-centre B cell-like diffuse large B cell lymphoma (GCB-DLBCL) and Burkitt lymphoma
8227	AKAP17A	A-kinase anchoring protein 17A	Yp11.2		A PAR1 gene encoding a protein kinase A anchoring protein of the spliceosome complex, involved in the regulation of alternate splicing in some mRNA precursors
438	ASMT	Acetylserotonin *O*-methyltransferase	Yp11.2	Asmt (chr Y, X)	A PAR1 gene encoding a member of the methyltransferase superfamily, involved in the final reaction in the synthesis of melatonin and meantonin-associated pathophysiology
207063	DHRSX	Dehydrogenase/reductase X-linked	Yp11.2	Dhrsx (chr 4)	A PAR1 gene encoding a non-classical secretary protein, associated with starvation induced autophagy
9189	ZBED1	Zinc finger BED-type containing 1	Yp11.2		A PAR1 gene encoding a nucleus protein, likely involved in regulating genes related to cell proliferation, highly expressed in gastric cancer
4267	CD99	CD99 molecule (Xg blood group)	Yp11.2	Cd99 (chr 4)	A PAR1 green encoding a cell surface glycoprotein involved in leukocyte migration, T-cell adhesion, ganglioside GM1 and transmembrane protein transport, and T-cell death by a caspase-independent pathway; associated with solitary firous tumor
6736	SRY	Sex determining region Y	Yp11.2	Sry (chr Y)	A MSY intronless gene encoding a protein harboring a high mobility group (HMG)-box, and is the founder of the SRY-box (SOX) family of transcription factors. SRY is the testis-determining factor (TDF), responsible for male sex determination during embryogenesis
6192	RPS4Y1	Ribosomal protein S4, Y-linked 1	Yp11.2	Rps4x (chr X)	A MSY gene encoding the ribosomal protein S4, a component of the 40S ribosomal subunit; upregulation could be associated with Parkinson’s disease and schizophrenia
7544	ZFY	Zinc finger protein, Y-linked	Yp11.2	Zfy1/Zhy2 (chr Y)	A MSY gene encoding a zinc finger transcription factor, likely involved in regulation of male germ cell differentiation
90655	TGIF2LY	TGFB induced factor homeobox 2 like Y-linked	Yp11.2	Tgif2lx1 (chr X)	A MSY gene encoding a member of the TALE/TGIF homeobox family of transcription factors, potential regulator of its X homologue
83259	PCDH11Y	Protocadherin 11 Y-linked	Yp11.2	Pcdh11x (chr X)	A MSY gene encoding a member of the protocadherin family, harboring an extracellular domain containing seven cadherin repeats, a transmembrane domain, and a cytoplasmic tail different from those of the classical cadherins, likely involved in cell-cell recognition and cerebral asymmetry during development of the central nervous system
7258	TSPY1	Testis specific protein, Y-linked	Yp11.2	Tspy-ps (pseudogene) (chr Y)	A testis-specific repetitive MSY gene encoding a protein harboring a SET/NAP domain, involved in binding to cyclin B-CDK1 complex, likely involved in spermatogonial germ cell renewal and male meiotic division. TSPY is a proto-oncogene for the gonadoblastoma locus on the Y chromosome (GBY), promoting cell proliferation. A X-located homologue, TSPX, possesses contrasting functions in cell cycle regulation and serves as a tumor suppressor
266	AMELY	Amelogenin, Y-linked	Yp11.2	Amelx (chr X)	A MSY gene encoding a member of the amelogenin family of extracellular matrix proteins, involved in biomineralization during tooth enamel development
90665	TBL1Y	Transducin beta like 1 Y-linked	Yp11.2	Tbl1x (Chr X)	A MSY gene encoding a member of the WD40 repeat-containing protein family, mediating protein–protein interactions, involved in signal transduction, RNA processing, gene regulation, vesicular trafficking, and cytoskeletal assembly; mutations associated with deafness and coarctation of the aorta
8287	USP9Y	Ubiquitin specific peptidase 9, Y-linked	Yq11.221	Usp9y (chr Y)	A MSY gene and a member of the peptidase C19 family encoding a protein similar to ubiquitin-specific proteases, involved in cleavage of the ubiquitin moiety from ubiquitin-fused precursors and ubiquitinylated proteins
8653	DDX3Y	DEAD-box helicase 3, Y-linked	Yq11.221	Ddx3y (chr Y)	A MSY gene and a member of the DEAD (Asp-Glu-Ala-Asp)-box RNA helicase family, involved in ATP binding, hydrolysis, RNA binding, and in the formation of intramolecular interactions; upregulation could be associated with Parkinson’s disease and schizophrenia
7404	UTY	Ubiquitously transcribed tetratricopeptide repeat containing, Y-linked	Yq11.221	Uty (chr Y)	A MSY gene encoding a protein containing tetratricopeptide repeats and homologous to an X-located lysine demethylase 6A (KDM6A), involved in modification of the lysine residues of histone H3; a candidate for the H-Y histocompatibility antigen, involved in graft rejection of male stem cells
9087	TMSB4Y	Thymosin beta 4, Y-linked	Yq11.221	Tmsb4x (chr X)	A MSY gene encoding an actin sequestering protein; likely a tumor suppressor in male breast cancer
9084	VCY	Variable charge Y-linked	Yq11.221		A MSY gene and a member of a family of human VCX/Y repetitive genes, specifically expressed in the testis with unknown functions
22829	NLGN4Y	Neuroligin 4, Y-linked	Yq11.221	Nlgn3 (chr X)	A MSY gene encoding a type I membrane protein and a member of the neuroligin family, which are cell adhesion molecules present at the postsynaptic side of the synapse and essential for the formation of functional synapses; could be associated with phenotypes of autism spectrum disorder
86614	HSFY1	Heat shock transcription factor, Y-linked 1	Yq11.222	Hsf2 (chr 10)	A double-copy MSY gene encoding a member of the heat shock factor (HSF) family of transcriptional activators for heat shock proteins
9083	BPY2	Basic charge Y-linked 2	Yq11.223		A MSY repetitive gene expressed specifically in testis. The encoded protein interacts with ubiquitin protein ligase E3A, involved in male germ cell development and male infertility
8284	KDM5D	Lysine demethylase 5D	Yq11.223	Kdm5d (chr Y)	A MSY gene encoding a histone demethylase containing zinc finger domains, a candidate for the H-Y antigen; involved in neurodevelopment, cardiovascular development and disease and various cancers
9086	EIF1AY	Eukaryotic translation initiation factor 1A, Y-linked	Yq11.223	Eif1a (chr 18)	A MSY gene encoding a protein related to eukaryotic translation initiation factor 1A (EIF1A), associated with dilated ischemic cardiomyopathy and infertility
5940	RBMY1A1	RNA binding motif protein, Y-linked, family 1, multiple members	Yq11.223	Rbmy (chr Y)	A testis-specific and multiple-copy MSY gene encoding a protein containing an RNA-binding motif in the N-terminus and four SRGY (serine, arginine, glycine, tyrosine) boxes in the C-terminus, involved in RNA splicing regulation during spermatogenesis; aberrantly expressed in liver and other cancers
9081	PRY2	PTPN13 like Y-linked 2	Yq11.223	Ptpn13 (chr 5)	A testis-specific MSY gene encoding a protein with a low similarity to protein tyrosine phosphatase, non-receptor type 13; could be involved in apoptosis
1617	DAZ1	Deleted in azoospermia 1	Yq11.223	Dazl (chr 17)	A multiple-copy MSY gene encoding an RNA-binding protein important for spermatogenesis. Microdeletion of gene clusters could be involved in infertility in men
9085	CDY1	Chromodomain Y-linked 2A	Yq11.23	Cdyl (chr 13)	A multiple-copy MSY gene encoding a protein containing a chromodomain and a histone acetyltransferase catalytic domain; involved in germ cell biology and infertility
10251	SPRY3	Sprouty RTK signaling antagonist 3	Yq12	Spry3 (chr X)	A PAR2 gene, potentially involved in neurogenesis and pathogenesis of autism
6845	VAMP7	Vesicle associated membrane protein 7	Yq12	Vamp7 (chr X)	A PAR2 gene encoding a transmembrane protein and a member of the soluble N-ethylmaleimidesensitive factor attachment protein receptor (SNARE) family, involved in fusion of transport vesicles to target members in endosomes and lysosomes
3581	IL9R	Interleukin 9 receptor	Yq12	Il9r (chr 11)	A gene located at the most terminus of PAR2, associated with asthma pathogenesis. It encodes a cytokine receptor for interleukin 9 (IL9), involved in JAK kinases and STAT proteins functions; could be involved in immune functions, multiphe scherosis and preeclampsia
Non-functional gene/pseudogenes on the human Y chromosome					
5616	PRKY	Protein kinase Y-linked pseudogene	Yp11.2		A MSY pseudogene similar to the protein kinase X-linked on the PAR1 of the X chromosome, with detectable transcripts in various tissues
9082	XKRY	XK related, Y-linked	Yq11.222		A MSY gene encoding a putative memberane transport protein similar to X-linked Kell blood group. Minimal detectable transcripts in any tissues examined so far, hence its functionality is uncertain
146126	TXLNGY	Taxilin gamma pseudogene, Y-linked	Yq11.222-Yq11.223		A MSY pseudogene with broad transcript expression in various tissues

^a^Adopted from the National Center for Biotechnology Information (NCBI) and PubMed databases [35]

Noticeably, several MSY genes, such as *SRY*, *TSPY* and *RBMY*, are exceptions to such dosage-sensitive functions. They are primarily expressed in the testis and serve vital functions in the differentiation and physiology of this male-specific organ [38–40]. Their counterparts on the X chromosome are expressed ubiquitously in numerous tissue/cell types and mostly at similar levels between the sexes (Additional file 1: Figure S1D) and are subjected to X-inactivation [28]. Further, the respective encoded proteins could possess structural divergence(s), suggesting that they might serve different functions in various biological systems [24, 25, 41]. Accordingly, expression of these testis-specific MSY genes in somatic tissues could exert male-specific effects on the respectively affected organs/cells. We surmise that low-level/spatiotemporal expression of these MSY gene(s) during development/physiology could produce normal differences between the sexes [5, 42, 43], and aberrant/high level expression could result in male biases in the pathogeneses of various human diseases [6, 27, 41]. In particular, *TSPY* and its X homologue *TSPX* (*TSPYL2*) evolved from the same ancestral gene but diverged structurally in their encoded proteins to process contrasting functions in cell cycle regulation and androgen receptor (AR) transactivation [44, 45]. They represent a pair of homologues on the sex chromosomes, which could oppose each other in various biological processes [41]. *TSPY* is specifically expressed in the testis (Additional file 1: Figure S1E) and could serve important functions in spermatogonia stem cell renewal and male meiosis [46]. It is located on and is the putative gene for the gonadoblastoma locus on the Y chromosome (GBY) [47, 48]. It is frequently and aberrantly activated in various cancers, including gonadoblastoma, testicular germ cell tumors, melanoma, liver, head and neck and prostate cancers and promotes cell proliferation [48, 49]. TSPY interacts with AR and stimulates the AR transactivation of its target genes [45]. Importantly, *TSPY* is an androgen-responsive gene, and hence *TSPY* and *AR* form a positive feedback loop in amplifying their respective biological functions in male-specific manners [41]. *TSPX* is widely expressed equally in both male and female tissues (Additional file 1: Figure S1D) and is subjected to X-inactivation [50]. It retards cell proliferation and suppresses AR transactivation activities [45]. Accordingly, *TSPY* is a Y-located proto-oncogene and *TSPX* is an X-located tumor suppressor at the two extremes of the human oncogenic spectrum respectively [41]. This peculiar situation raises some very interesting scenarios on the roles of the X–Y homologues in cancers. As a proto-oncogene, abnormal activation of *TSPY* in somatic tissues could promote oncogenesis while an inactivation/deletion of *TSPX* could impair its tumor suppression function(s) [51, 52] specifically for males with only one X chromosome. If under certain conditions, *TSPX* escapes X-inactivation, it could increase the tumor suppression functions in females [29, 50]. Collectively, such aberrations could disproportionally exacerbate cancer initiation and progression in males.

Mosaic loss of the Y chromosome in 5–15% of the leukocytes of men represents one of the most common genetic abnormalities in humans [13–16, 18]. Since the Y chromosome includes the pseudoautosomal and male-specific regions, the loss of the PAR genes and those on MSY with dosage-sensitive functions could result in gene dosage deficiency in various biological systems. On the other hand, the testis-specific MSY genes are important for male-specific functions, and their loss might be less critical in such mLOY-mediated disease predispositions, except those associated with reproductive tissues. However, the abnormal expression of these-testis specific MSY genes in non-gonadal cells could modify the normal differentiation and physiology as well as the pathogenesis of the affected cells/tissues in male biased manners. Accordingly, there are two general categories of Y chromosome genes, those on the PARs and MSY with dosage-sensitivity and MSY genes with male/testis-specific expression and functions. Understanding the biology and genetics of individual genes on the Y chromosome could provide some clues on which genes are likely to be important for the maintenance of homeostasis and their losses or aberrant activations could contribute to disease predisposition in men.

## Of mice and men

The laboratory mouse has been widely used as experimental models for human diseases using transgenic means [53, 54], including male and female animals with different sex chromosome constitutions [55, 56]. Beside sex determination, the genetic contents of the human and mouse Y chromosome are quite distinct [10, 57]. The mouse Y chromosome is ~ 92 Mb in size, likely one of the largest and most gene-rich Y chromosomes in mammals. Its PAR is ~ only 700 kb, located at the telomere of the long arm, and harbors only 3 genes, i.e. *Asmt*, *Sts* (pseudogene) and *Mid1*. The mouse MSY is > 90 Mb in size and is entirely euchromatic. It harbors ~ 700 genes, but only 8 ancestral genes, i.e. *Sry*, *Zfy1*/*Zfy2*, *Usp9y*, *Ddx3y*, *Uty*, *Kdm5d*, *Tspy (*pseudogene*)*, and *Rbmy*, are conserved on the human Y chromosome (Table 1). Other orthologues of the human Y chromosome genes are located on either the X chromosome or autosomes of the mouse. The remainder ~ 690 genes of the mouse MSY are acquired repetitive genes, some of which are embedded in a 500-kb repeat unit, likely to be important for mouse fertility, but are not conserved in any mammalian Y chromosome [57]. Significantly, only two MSY genes, i.e. *Sry* (the sex-determining gene) and *Eif2s3y* (absent on the human Y chromosome), are sufficient to generate male mice with spermatogenesis [58]. Further, these two MSY genes could be replaced by the activation of *Sox9* (the *Sry* downstream gene [26]) and *Eif2s3x* (the X-homologue of *Eif2s3y*) to produce similarly functional male mice without a Y chromosome [59]. Although transgenic mouse studies have provided critical information on the functional aspects on some of those conserved MSY genes [54, 58–61], studies on the other MSY genes of the human Y chromosome with the laboratory mouse might require some retrofitting of the mouse Y chromosome. In particular, the mouse *Tspy* is a recently evolved pseudogene, harboring numerous in-frame mutations disrupting its open reading frame. Transgenic studies had generated a specific line, TgTSPY9, which harbors ~ 50 copies of the an 8.2-kb human *TSPY* structural gene tandemly integrated on the Y chromosome of the mouse [62]. Since *TSPY* is tandemly repeated 30–60 times on the human Y chromosome [40], such tandem integration of the human transgene on the mouse Y chromosome resembles the organization of the human endogenous gene. Characterization of animals from this transgenic line showed that the human *TSPY* transgene is expressed in similar patterns as those of the human endogenous gene, including normal expression in spermatogonia and spermatocytes in the testis [62] and aberrant activation during oncogenesis [63]. Hence, TgTSPY9 is a humanized mouse model for *TSPY* gene. Accordingly, with the recent advances of genome editing and transgenic technologies [64–67], targeting the integration of human MSY gene(s) onto the mouse Y chromosome could be viable strategies to model and study their functions in biological processes, involved in various aspects of development, physiology and disease pathogeneses.

## Supplementary information

**Additional file 1: Figure S1.** Examples of expression patterns of Y chromosome genes in 54 normal male (blue) and female (red) human tissues in the Genotype-Tissue Expression (GTEx) Project database, analyzed on 05/13/2020.

**Additional file 2: Table S1.** Diseases and pathways associated with Y chromosome genes revealed by gene ontology analysis.

## Data Availability

Not applicable.
