# Supplementary material for: Y chromosome in health and diseases
Source: Cell Biosci. 2020 Aug 13;10:97. doi: 10.1186/s13578-020-00452-w (PMC7427063; doi:10.1186/s13578-020-00452-w)
Supplement: Supplementary file 1 — Additional file 1: Figure S1. Examples of expression patterns of Y chromosome genes in 54 normal male (blue) and female (red) human tissues in the Genotype-Tissue Expression (GTEx) Project database, analyzed on 05/13/2020. [file 13578_2020_452_MOESM1_ESM.pdf]

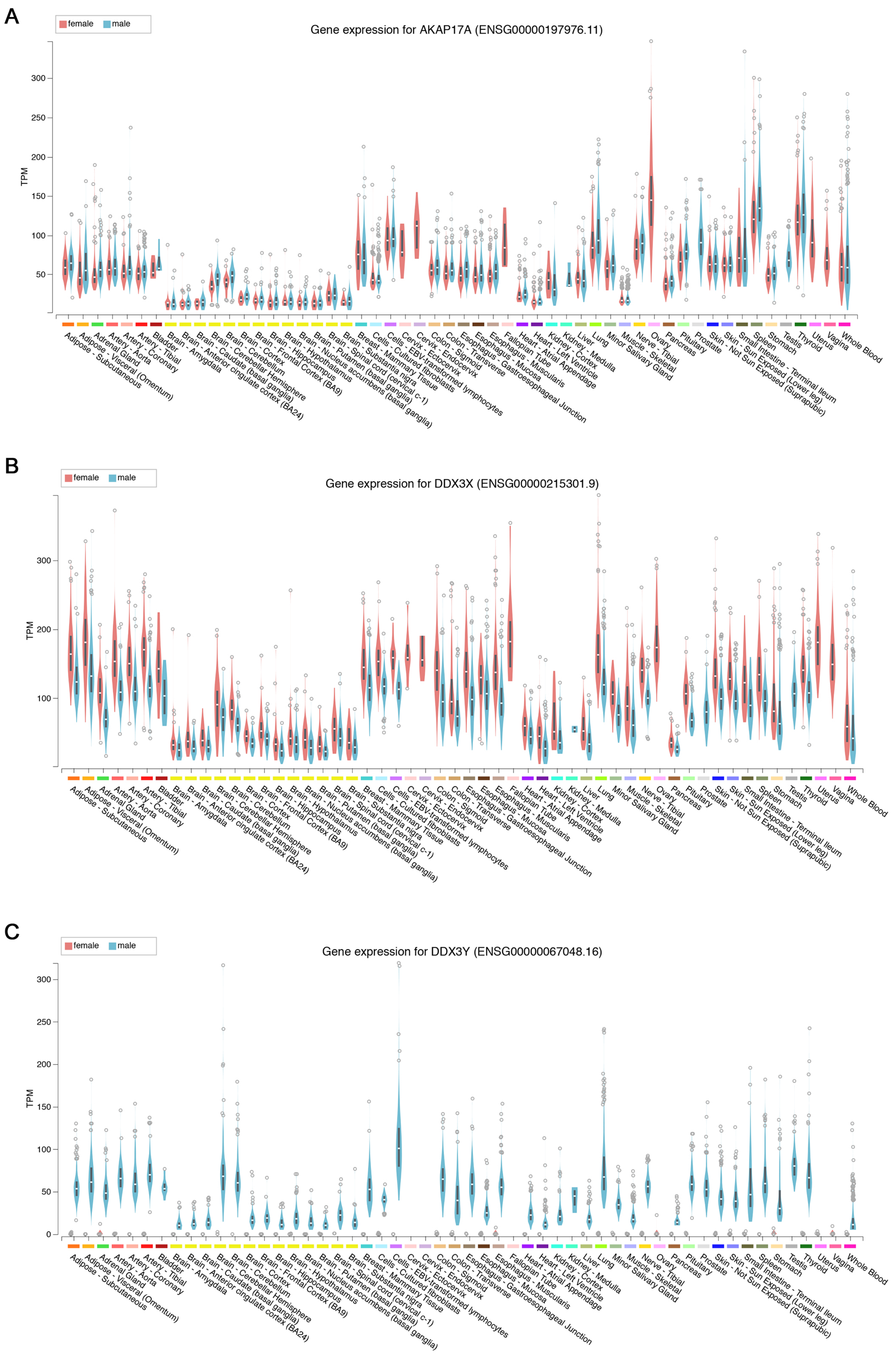

A
female
male
Gene expression for AKAP17A (ENSG00000197976.11)
TPM
300
250
200
150
100
50
Adipose - Subcutaneous
Adipose - Visceral (Omentum)
Adrenal Gland
Artery - Aorta
Artery - Coronary
Artery - Tibial
Bladder
Brain - Amygdala
Brain - Anterior cingulate cortex (BA24)
Brain - Caudate (basal ganglia)
Brain - Cerebellar Hemisphere
Brain - Cerebellum
Brain - Cortex
Brain - Frontal Cortex (BA9)
Brain - Hippocampus
Brain - Hypothalamus
Brain - Nucleus accumbens (basal ganglia)
Brain - Putamen (basal ganglia)
Brain - Spinal cord (cervical c-1)
Brain - Substantia nigra
Breast - Mammary Tissue
Cells - Cultured fibroblasts
Cells - EBV-transformed lymphocytes
Cervix - Ectocervix
Cervix - Endocervix
Colon - Sigmoid
Colon - Transverse
Esophagus - Gastroesophageal Junction
Esophagus - Mucosa
Esophagus - Muscularis
Fallopian Tube
Heart - Atrial Appendage
Heart - Left Ventricle
Kidney - Cortex
Kidney - Medulla
Liver
Lung
Minor Salivary Gland
Muscle - Skeletal
Nerve - Tibial
Ovary
Pancreas
Pituitary
Prostate
Skin - Not Sun Exposed (Suprapubic)
Skin - Sun Exposed (Lower leg)
Small Intestine - Terminal Ileum
Spleen
Stomach
Testis
Thyroid
Uterus
Vagina
Whole Blood
B
female
male
Gene expression for DDX3X (ENSG00000215301.9)
TPM
300
200
100
Adipose - Subcutaneous
Adipose - Visceral (Omentum)
Adrenal Gland
Artery - Aorta
Artery - Coronary
Artery - Tibial
Bladder
Brain - Amygdala
Brain - Anterior cingulate cortex (BA24)
Brain - Caudate (basal ganglia)
Brain - Cerebellar Hemisphere
Brain - Cerebellum
Brain - Cortex
Brain - Frontal Cortex (BA9)
Brain - Hippocampus
Brain - Hypothalamus
Brain - Nucleus accumbens (basal ganglia)
Brain - Putamen (basal ganglia)
Brain - Spinal cord (cervical c-1)
Brain - Substantia nigra
Breast - Mammary Tissue
Cells - Cultured fibroblasts
Cells - EBV-transformed lymphocytes
Cervix - Ectocervix
Cervix - Endocervix
Colon - Sigmoid
Colon - Transverse
Esophagus - Gastroesophageal Junction
Esophagus - Mucosa
Esophagus - Muscularis
Fallopian Tube
Heart - Atrial Appendage
Heart - Left Ventricle
Kidney - Cortex
Kidney - Medulla
Liver
Lung
Minor Salivary Gland
Muscle - Skeletal
Nerve - Tibial
Ovary
Pancreas
Pituitary
Prostate
Skin - Not Sun Exposed (Suprapubic)
Skin - Sun Exposed (Lower leg)
Small Intestine - Terminal Ileum
Spleen
Stomach
Testis
Thyroid
Uterus
Vagina
Whole Blood
C
female
male
Gene expression for DDX3Y (ENSG00000067048.16)
TPM
300
250
200
150
100
50
0
Adipose - Subcutaneous
Adipose - Visceral (Omentum)
Adrenal Gland
Artery - Aorta
Artery - Coronary
Artery - Tibial
Bladder
Brain - Amygdala
Brain - Anterior cingulate cortex (BA24)
Brain - Caudate (basal ganglia)
Brain - Cerebellar Hemisphere
Brain - Cerebellum
Brain - Cortex
Brain - Frontal Cortex (BA9)
Brain - Hippocampus
Brain - Hypothalamus
Brain - Nucleus accumbens (basal ganglia)
Brain - Putamen (basal ganglia)
Brain - Spinal cord (cervical c-1)
Brain - Substantia nigra
Breast - Mammary Tissue
Cells - Cultured fibroblasts
Cells - EBV-transformed lymphocytes
Cervix - Ectocervix
Cervix - Endocervix
Colon - Sigmoid
Colon - Transverse
Esophagus - Gastroesophageal Junction
Esophagus - Mucosa
Esophagus - Muscularis
Fallopian Tube
Heart - Atrial Appendage
Heart - Left Ventricle
Kidney - Cortex
Kidney - Medulla
Liver
Lung
Minor Salivary Gland
Muscle - Skeletal
Nerve - Tibial
Ovary
Pancreas
Pituitary
Prostate
Skin - Not Sun Exposed (Suprapubic)
Skin - Sun Exposed (Lower leg)
Small Intestine - Terminal Ileum
Spleen
Stomach
Testis
Thyroid
Uterus
Vagina
Whole Blood

D

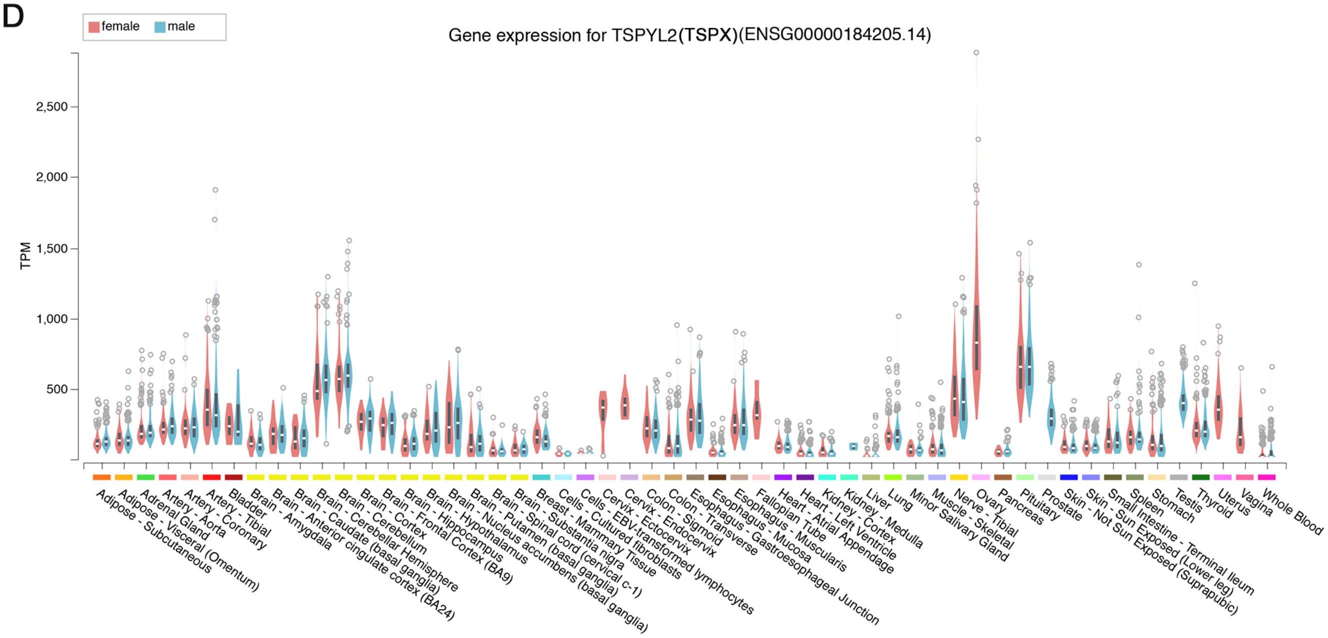

E

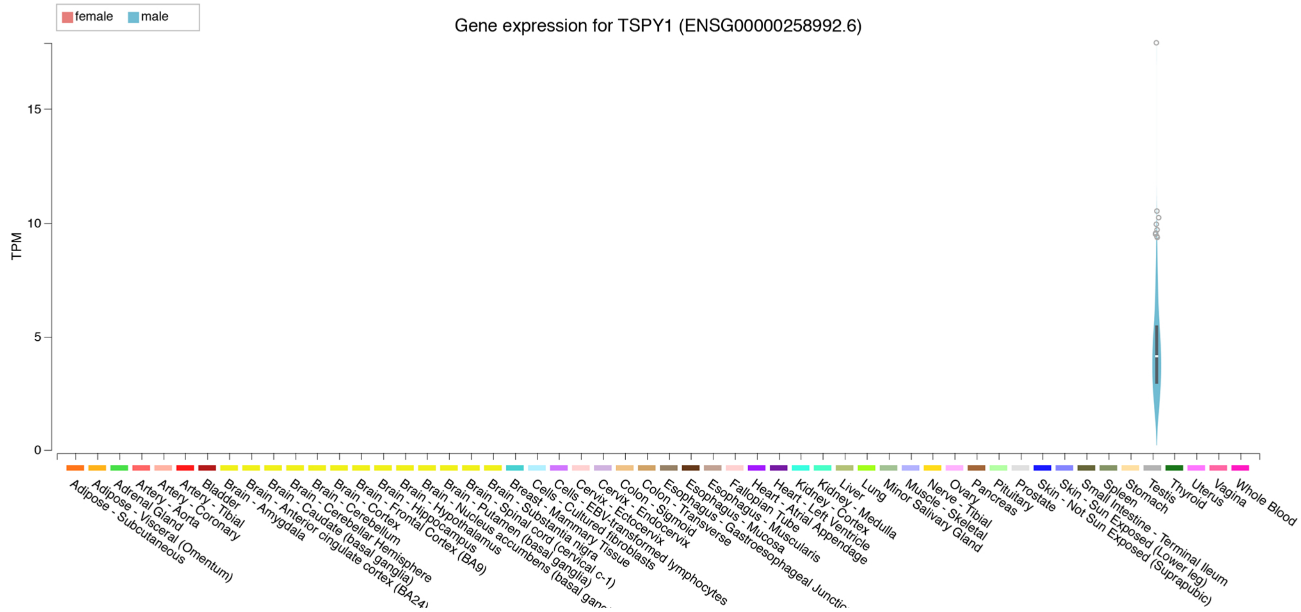

Figure S1. Examples of expression patterns of Y chromosome genes in 54 normal male (blue) and female (red) human tissues in the Genotype-Tissue Expression (GTEx) Project database, analyzed on 05/13/2020. A) AKAP17A is a PAR1 gene that escapes X-inactivation and is expressed in similar levels between the sexes. B) DDX3X is the X-homologue of the MSY DDX3Y gene. DDX3X and DDX3Y encode highly conserved proteins and likely serve similar biological functions in RNA splicing and/or translation processes. DDX3X escapes X-inactivation and is expressed differentially between female and male, as reflected by the respective number of X chromosome. C) The deficiency of DDX3X expression in male is compensated by the specific DDX3Y expression from the Y chromosome. D) TSPX (TSPYL2) is the single-copy X-homologue of the MSY repeated TSPY gene. They have evolutionarily diverged and likely serve distinct biological functions. TSPX is subjected to X-inactivation and is expressed in similar levels between the sexes. E) TSPY is specifically expressed in the testis and likely serves vital functions in the differentiation and physiology of this male-specific organ.
